# Neuropilin-1 is a receptor for extracellular miRNA and AGO2/miRNA complexes and mediates the internalization of miRNAs that modulate cell function

**DOI:** 10.18632/oncotarget.10929

**Published:** 2016-07-29

**Authors:** Gerald J. Prud'homme, Yelena Glinka, Zsuzsanna Lichner, George M. Yousef

**Affiliations:** ^1^ Keenan Research Centre for Biomedical Science, St. Michael's Hospital, Toronto, ON M5B 1W8, Canada; ^2^ Department of Laboratory Medicine, St. Michael's Hospital, Toronto, ON M5B 1W8, Canada; ^3^ Department of Laboratory Medicine and Pathobiology, University of Toronto, Toronto, ON M5G 1L5, Canada

**Keywords:** miRNA, endocytosis, endothelial cells, neuropilin, renal cell carcinoma

## Abstract

Extracellular miRNAs are increasingly studied as markers for specific diseases. They are released in biological fluids in a remarkably stable form, and may play a role in intercellular communication. They are thought to be protected against degradation by either encapsulation within microparticles, or by binding to proteins (mostly AGO2). The particulate forms may be internalized by endocytosis or membrane fusion, but the protein-bound forms require a receptor mechanism for their uptake. A major question is whether there are natural cell-membrane receptors that capture and internalize protein-bound functional miRNAs. We examined neuropilin-1 (NRP1), in view of its properties as a receptor for many ligands, including growth factors such as vascular endothelial growth factor (VEGF), and efficiency at mediating ligand internalization. It is expressed by endothelial cells, many other normal cell types, and cancer cells. Here, we report that NRP1 binds miRNAs with high affinity, and promotes their entry into the cell. Furthermore, the internalized miRNAs remain functional, as they specifically regulate proliferation and migration of cancer cells, as well as tube formation by human endothelial cells. Anti-NRP1 antibodies or NRP1 siRNA knockdown block miRNA effects, further confirming NRP1-mediated uptake. VEGF does not compete with miRNAs for binding to NRP1. In addition, NRP1 binds extracellular AGO2 (carrying miRNA or not), and internalizes AGO2/miRNA complexes. Because miRNA bound to AGO2 appears to the most abundant form in body fluids, this may have important physiological and pathological effects.

## INTRODUCTION

miRNAs are short non-coding RNAs that are estimated to regulate up to two-thirds of all human genes. They have also been reported to play important roles in tumor development and metastasis [1]. Extracellular miRNAs are found in biological fluids [2]. They might play a role in inter-cellular communication, but this remains unclear. Unlike other types of RNA, miRNAs are quite stable in the blood. They appear to be protected against degradation either by encapsulation within microparticles, or because they are bound to proteins (mostly AGO2) in a non-particulate form. While the encapsulated forms may be internalized by endocytosis or membrane fusion, the protein-bound forms require a mediator or receptor facilitating transport across the membrane. Several important publications implicate protein complexes in this process. For instance, Wagenaar et al. demonstrated productive uptake and endosomal sorting of oligonucleotides, but the mechanism of cell membrane penetration was not determined [3]. Vickers et al. demonstrated the role of high-density lipoproteins (HDL), and its receptor SR-BI, in non-exosomal transport and uptake of a number of miRNAs with functional targeting capabilities [4]. However, only a small fraction of circulating miRNA was complexed to HDL, and this mechanism may not be dominant [2].

The involvement of specific miRNAs in kidney cancer pathogenesis has been documented in the literature [5, 6]. More recently, the role of miRNAs in mediating communication between cancer cells and endothelial cells has been postulated [5, 7]. A major question is whether there are natural receptors for extracellular miRNAs, which would facilitate their entry into cells. Toll-like receptors (TLRs) can bind miRNA and other RNA species, but the RNA does not appear to be transported across the cell membrane [8]. Several publications describe artificial systems for miRNA delivery, such as cell-penetrating peptides (CPPs) and various types of nanoparticles [9]. However, these synthetic systems are not likely to mimic the natural mechanisms of RNA exchange between cells.

We hypothesized that there is a high affinity receptor for miRNAs on cells, with the capacity to internalize their ligand. In view of its properties as a multispecific receptor [10], and efficient mediator of peptide internalization [11], we examined neuropilin-1 (NRP1) as a potential miRNA receptor. As we have reviewed [10], NRP1 is a multifunctional protein with numerous ligands [12–14], and only salient features are mentioned here. It is a coreceptor for several growth factors and other mediators, including vascular endothelial growth factor (VEGF), transforming growth factor β1 (TGF-β1), hepatocyte growth factor (HGF) and platelet-derived growth factor (PDGF). Interestingly, in most cases NRP1 interacts with both the soluble growth factor and its receptors, and generally enhances responses. As such it plays a physiological and pathological role in many situations, including angiogenesis, wound healing, cancer and immunity. It is expressed by a variety of cell types; notably endothelial cells and cancer cells. It is a coreceptor for the class 3 semaphorins (SEMA3), which play a key role in axonal guidance. It interacts with integrins, and is involved in epithelial-to-mesenchymal transition (EMT), Hedgehog signaling, and the survival of stem cells. The molecular basis for these numerous interactions is not well understood, but crystal structure studies and other investigations have identified the binding sites of VEGF. Importantly, it binds negatively charged heparin [15, 16], which raised the possibility that it might also bind nucleic acids. Here, we report that NRP1 binds miRNAs with high affinity and promotes their entry into the cell. Furthermore, the internalized miRNAs are functional. We also report that NRP1 binds AGO2 and AGO2/miRNA complexes. Because miRNA bound to AGO2 is possibly the most abundant form in body fluids, this may have important physiological consequences [17].

## RESULTS

### Recombinant NRP1 binds synthetic miRNA oligonucleotides

**Figure 1:**
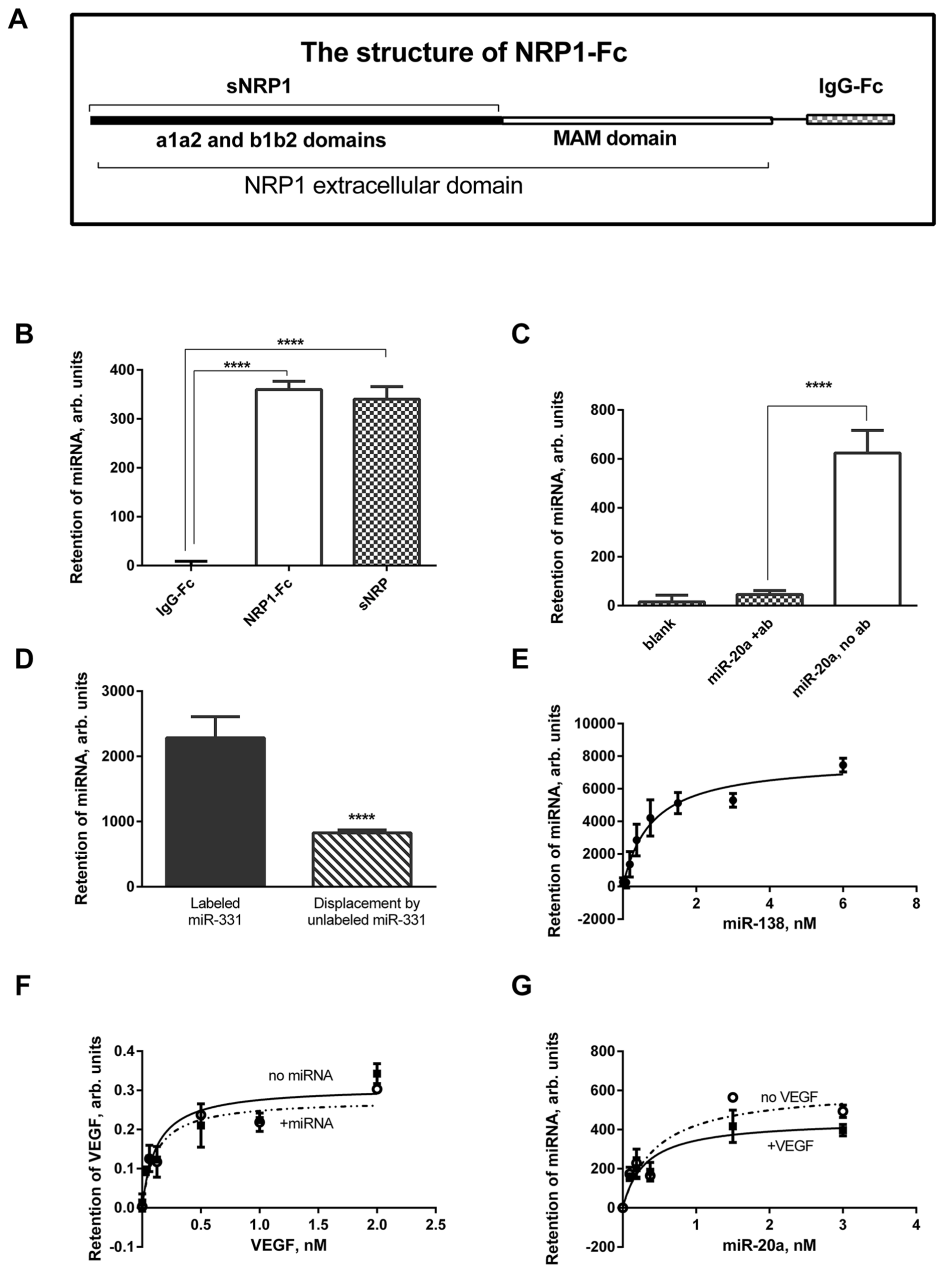
Recombinant NRP1 binds miRNA mimics **A.** The structure of the NRP1-Fc, lacking the transmembrane and cytoplasmic portions of NRP1, and truncated sNRP1 consisting only of the a1a2 and b1b2 domains. **B.** miR-331 is retained on the NRP1-Fc- and sNRP1-coated plate but is not captured by the Fc-tag protein. **C.** Pre-treatment of the sNRP1-coated plate with anti-NRP1 antibody (ab) prevents binding of miR-20a to the plate. **D.** miR-331 bound to the sNRP-coated plate (left bar) was displaced during the overnight incubation with unlabeled miRNA (right bar). **E.** A typical binding curve: miR-138 is bound to the sNRP-coated plate in the presence of 0.9 mM Mg and 1.26 mM Ca (HBSS with Ca^2+^ and Mg^2+^). **F.** 1 nM miR-20a does not modulate the binding of VEGF to sNRP1. **G.** 1 nM VEGF insignificantly affects the affinity of miR-20a to sNRP1. The data is representative of 3-6 independent experiments. In all cases, the data is presented as the Mean±SEM; **** P<0.0001.

Here we demonstrate that synthetic miRNAs bind to recombinant NRP1 with high affinity. The panel of miRNAs included miR-20a, miR-138, miR-331, miR-422, and control RNA, which were biotinylated using the Pierce biotinylation kit. The first two are reported to be deregulated in kidney cancer [15, 18]. Both full-length NRP1-Fc and a truncated molecule sNRP1 (Figure 1A) bound denatured miR-331 (Figure 1B). The IgG-Fc-tag alone did not bind miRNA. This indicates that the miRNA binding site(s) is/are located within either a1a2 or b1b2 domains of NRP1, or possibly both domains. The use of either sNRP1 or NRP1-Fc is indicated in the Legends. An anti-NRP1 antibody blocked the miRNA binding (Figure 1C). For blocking, we used monoclonal antibody MNRP1685A (from Genentech), which binds to the b1b2 domain of NRP1 of both human and mouse. This suggests that the b1b2 domains are the primary site of binding, although further investigations are required. Bound miRNA was displaced by non-labeled miRNA (Figure 1D) or heparin (not shown). All this confirms that NRP1 specifically binds miRNAs. MiRNA denaturation did not change affinity, and because of this denaturation was omitted from the protocol in subsequent work. Since miRNA mimics are double-stranded, it is clear that both single-stranded and double-stranded miRNA can bind to NRP1.

A typical binding curve of non-denatured miRNA binding is presented on Figure 1E, and the dissociation constants for other miRNAs are listed in Table 1. The binding fits a single-site model, although additional binding sites with different affinity might be observed with other miRNA species or in a wider range of concentrations. The binding affinity of NRP1-Fc and sNRP1 was very close (Table 1). An important factor modulating the binding affinity is the concentration of divalent cations (Table 1). Indeed, Mg^2+^ and Ca^2+^ altered the affinity of miRNAs, but binding occurred at physiological concentrations of these cations. Notably, addition of Mg^2+^ alone increased binding affinity.

**Table 1:** Binding affinity of miRNAs to sNRP1 and NRP1-Fc and the effect of cations

miRNAxs	Immobilized protein	Kd, nM (no Mg, no Ca^2+^)	Kd, nM (0.5 mM Mg, no Ca^2+^)	Kd, nM (1.5 mM Mg, no Ca^2+^)	Kd, nM (at 0.9 mM Mg+1.2 mM Ca^2+^)
miR-20a	sNRP1		0.02823±0.00975		0.3864±0.02742
miR-138	sNRP1		0.3484±0.0814		0.7720±0.07681
miR-331	sNRP1	1.2±0.7	0.03830±0.01483	Kd=0.071±0.025	0.8500±0.1688
miR-331	NRP1-Fc				0.9112±0.3860
miR-422	sNRP1		0.1375±.02458		0.3441±0.0657

*The data is representative of 6 independent experiments.

The affinity of a miRNA to NRP1 may be modulated in the presence of its other ligands, such as VEGF, also present in the circulation. We address these issues in Figure 1F, 1G. We compared the binding of miRNA in the presence or absence of pre-bound VEGF, and did not find a difference within the range of concentrations used (Figure 1G). Similarly, pre-bound miRNA insignificantly affected the affinity of VEGF to its receptor (Figure 1F).

While positively-charged motifs in NRP1 are likely essential for binding of the negatively-charged miRNA, the role of other factors such as protein conformation is not clear. To examine the role of these factors, we tested the binding of miRNA to a plate coated with poly-ornithine instead of NRP1. The concentration of this positively-charged polypeptide was the same as that of sNRP1. The Kd value acquired in this experiment was 6.668±1.293. This is an order of magnitude higher than the values recorded for the binding to sNRP1 (Table 1). The relatively lower affinity despite the very high net positive charge suggests that the other factors, yet to be determined, make a significant contribution to the high affinity of miRNAs to NRP1.

The experiments described above demonstrate that NRP1 is an RNA-binding protein, which can capture miRNAs with high affinity under physiologically relevant conditions, i.e., non-denatured RNA, neutral pH, physiological concentrations of calcium and magnesium, 37°C.

### Naked miRNA binds to and is internalized by NRP1-expressing cancer cells in vitro

NRP1 is found both on the cell surface of a number of cancer cells, and can be rapidly internalized [11, 19]. We hypothesized that miRNAs bound to NRP1 can be translocated across the cell membrane. Here, we present evidence that miRNAs in fact bind to natural NRP1 on the cell surface and translocate into the cytoplasm of renal clear carcinoma cells.

**Figure 2:**
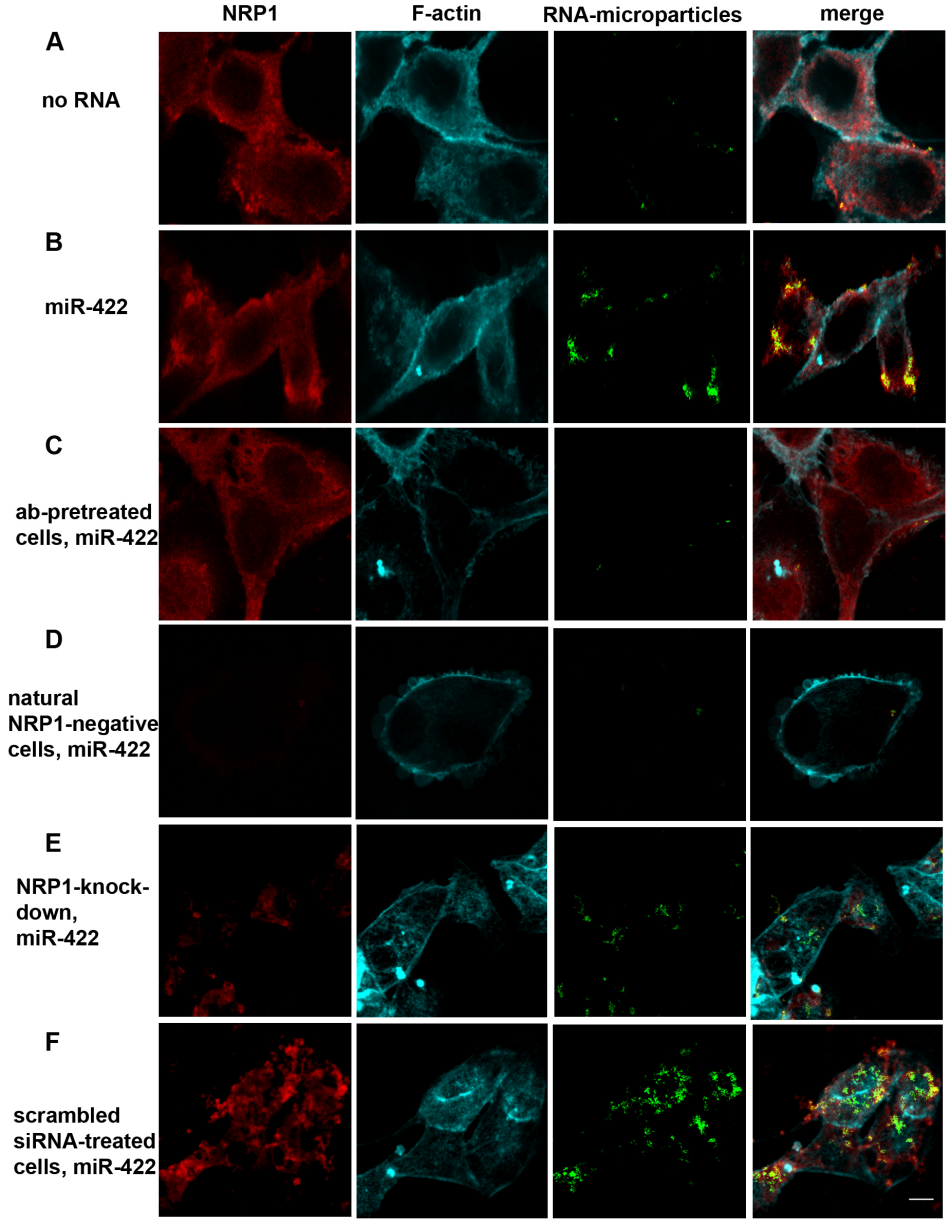
Biotinylated miR-422 conjugated to fluorescent streptavidin-coated microparticles binds to NRP1 expressed by ACHN cells and translocates into the cytoplasm The uptake of the beads is visualized by confocal microscopy. The fluorescent beads (green) are found in z- sections cutting through the nucleus. They co-localize with NRP1 (red; see also Figure 3). Cells are counter-stained for f-actin (cyan). **A.** Spontaneous uptake of the unconjugated beads is very low. B. Unassisted uptake of miR-422 conjugated to the beads by ACHN cells. **C, D.** Cells pre-treated with a blocking anti-NRP1 antibody (ab), or natural NRP1-negative cells, uptake negligible amount of miR-422-bead conjugate. **E, F.** Similarly, knockdown of NRP1 by siRNA severely depressed bead uptake, as compared to the scrambled siRNA control. Cells were transfected with siRNA using the unassisted translocation protocol. The data is representative of 6 independent experiments.

786-O and ACHN cells both express NRP1 as determined by flow cytometry analysis (not shown). To demonstrate translocation we conjugated the biotinylated miRNAs used in the cell-free binding assays with streptavidin-coated fluorescent microparticles, and incubated them with ACHN kidney cancer cells pre-treated or not with a blocking anti-NRP1 antibody (Figure 2A-2C). Fluorescent microparticles were found on the cell membrane and in the cytoplasm. MiRNA notably increased the number of the internalized particles, and the anti-NRP1 antibody decreased it to the level of the negative control. NRP1-negative cells (a small fraction of ACHN cells) did not uptake miRNA under the same treatment conditions (Figure 2D). In addition, siRNA knockdown of NRP1 caused severe inhibition of bead uptake whereas sham siRNA was not inhibitory (Figure 2E, 2F). Note that the cells were treated with siRNA without a transfection agent and, nevertheless, NRP1 was knocked down as described further below.

**Figure 3:**
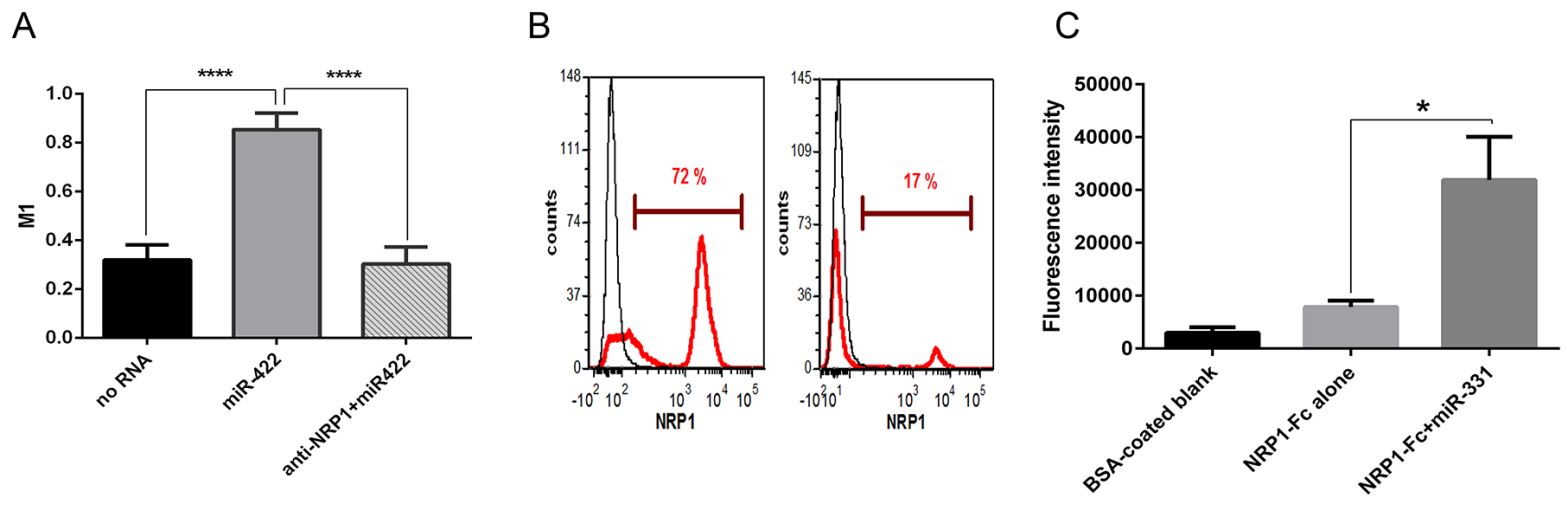
NRP1 colocalization with miRNA conjugated to the beads, unassisted NRP1 knockdown, and binding control **A.** Colocalization index. The experiment was performed as in Figure 2. In the cytoplasm 90% of miR-422 is colocalized with NRP1 as estimated by the calculation of Manders' coefficient (M1), and this was blocked by anti-NRP1 antibody. The data represents the Mean±SEM; **** P < 0.0001. **B.** NRP1-positive BT-474 breast cancer cells treated with NRP1-targeting siRNA without transfection reagent express significantly less NRP1 than the cells treated with scrambled siRNA. After 3 days in culture the cells were fixed, permeabilized, stained for NRP1, and analyzed by flow cytometry. The data is representative of three independent experiments. **C**. The streptavidin-coated microbeads did not bind significantly to NRP1-Fc, but binding was enhanced by pre-incubating the NRP1-Fc coated plate with a biotin-conjugated miRNA. Retention of the beads was quantified by fluorescent ELISA. Three experiments yielded similar results; the data represents Mean±SEM, *. P < 0.05.

Particle-associated fluorescence was co-localized with NRP1. We used ImageJ to quantify the colocalization. About 90% of the particle-associated fluorescence was co-localized with NRP1 in cytoplasm when the particles were conjugated with miRNA, while this value was only about 25% in the negative control and in the antibody-pretreated cells (Figure 3A). This indicates that extracellular miRNAs can bind to NRP1 and translocate across the cell membrane in a NRP1-dependent way.

We also confirmed this observation in an independent experiment. For this, we incubated NRP1-positive BT-474 breast carcinoma cells with NRP1-targeting siRNA in a serum-free medium without transfection reagent and observed the knockdown of the targeted protein (Figure 3B). The marked reduction of the expression indicated that the unassisted transfection was productive, as mentioned above for renal carcinoma cells.

As noted above, the streptavidin-coated microparticles had a limited ability to bind to the cells in the absence of miRNA. To exclude the possibility that these particles directly bind to NRP1 irrespective of miRNA, we performed a cell-free binding assay (Figure 3C). It shows that the microparticles did not have significantly more affinity for plate-bound NRP1-Fc than to the blank control (BSA only). However, when plate-bound NRP1-Fc was preincubated with biotin-conjugated miRNA the binding of the beads increased markedly. Therefore, we conclude that the binding of the fluorescent microparticles to the cells was not due to affinity to NRP1 by itself, but rather to NRP1/miRNA.

### NRP1 binds AGO2 with or without miRNA

**Figure 4:**
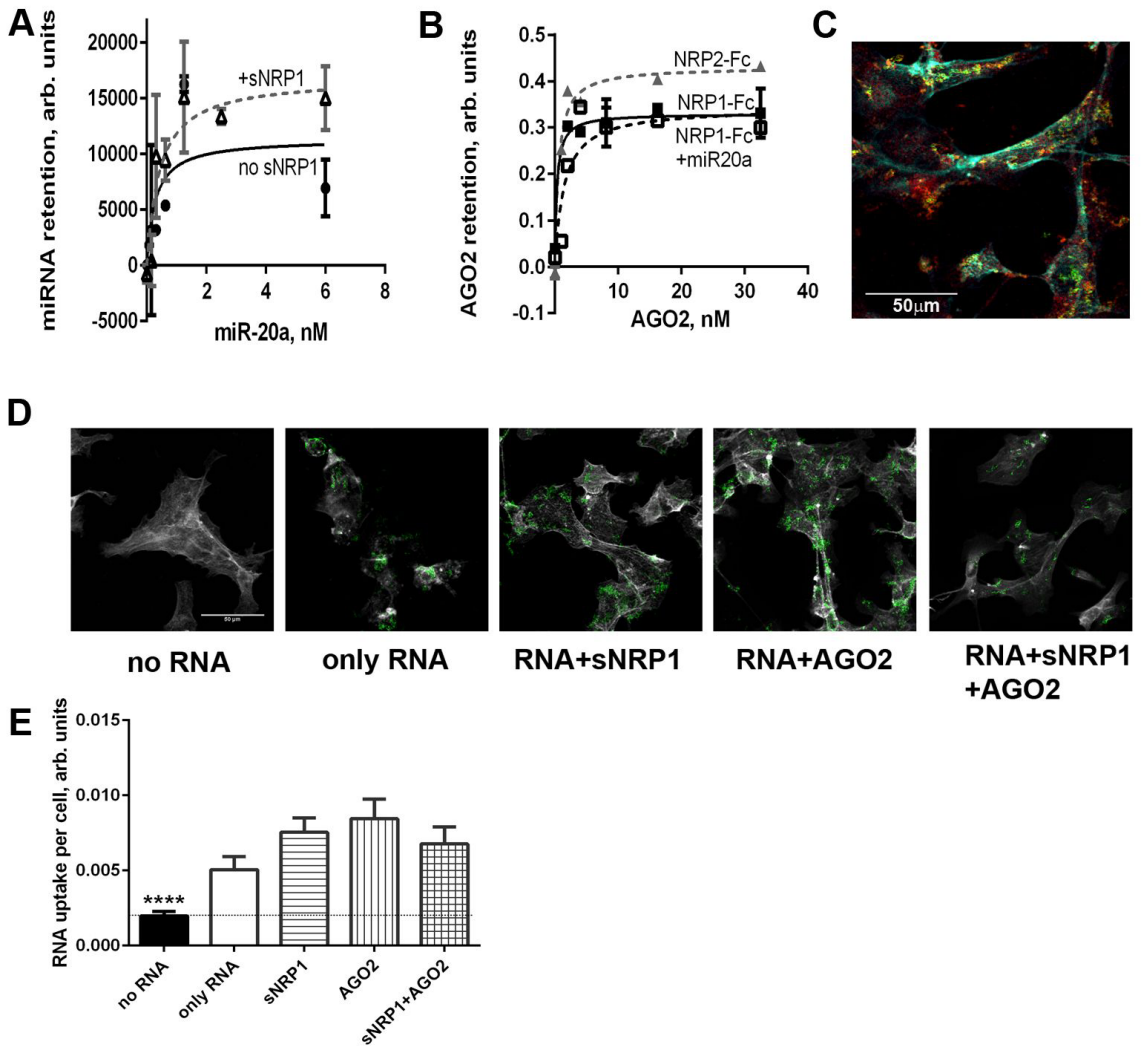
Recombinant AGO2 protein interacts with NRP1 and promotes miRNA uptake by ACHN cells **A.** miR-20a, pre-complexed or not with 1 nM sNRP1, binds to the plate coated with the Piwi domain of AGO2. Binding is expressed in relative luminescence units (RLU) after the subtraction of non-specific binding (arbitrary units). **B.** Full- length AGO2 binds to the immobilized NRP1-Fc. AGO2 is pre-mixed or not with the equimolar amount of miR-20a and serially diluted after 1 h incubation. Retention of AGO2 is quantified with anti-AGO2 antibody and visualized with TMB substrate. Binding is expressed as OD_450_-non-specific binding. All binding assays are performed in the presence of Ca^2+^ and Mg^2+^. **C.** ACHN cells internalize miR-331-AGO2 complex conjugated to streptavidin-coated fluorescent beads. Biotin-labeled miR-331 was preincubated with Piwi domain of AGO2 in an equimolar ratio and conjugated to the beads. The internalization of this complex by ACHN cells was observed by confocal microscopy. The beads (green) co-localize with NRP1 (red). Cells are counter-stained for f-actin (cyan). **D.** Interaction of miRNA with sNRP1, AGO2, or both proteins, facilitates the uptake of miR-331 by ACHN cells. Biotin-labeled miR-331 was preincubated or not with sNRP, AGO2-Piwi, or an equimolar mix of both proteins and conjugated to the beads as in panel C. miR-331 conjugated to the fluorescent streptavidin-coated beads (green) is seen in z-sections cutting through cytoplasm. The cell boundaries are delineated by f-actin staining (grey). **E.** The uptake of miRNA as shown in panel D is quantified as mean green fluorescence per cell using ImageJ. The data is presented as Mean±SEM; **** P<0.0001 versus all other bars. The data is representative of two independent experiments.

Circulating miRNAs are found in a complex with AGO2 protein which is believed to protect them from degradation. AGO2 contains an RNA-binding domain (Piwi). We found that full-length AGO2 binds miR-20a with an affinity that is lower than that of NRP1: Kd =7.272±4.692 nM and 0.3864±0.02742 nM, respectively. Pre-treatment of miRNA with an equimolar amount of sNRP1 did not prevent the retention of miRNA by the plate-immobilized Piwi domain of AGO2 (Figure 4A). Similarly, in the reversed setting, pre-incubation with AGO2 did not prevent retention of miRNA to the NRP1-coated plate.

Furthermore, full-length AGO2 by itself bound to NRP1-Fc with high affinity, and miRNA neither was required for this binding nor inhibited it (Figure 4B). The truncated AGO2 protein containing the Piwi domain bound NRP1 with the same affinity as the full-length protein (data not shown). AGO2 (Piwi) did not prevent the uptake of miRNA-conjugated beads by NRP1-positive cells, or block miRNA colocalization with NRP1 (Figure 4C). On the contrary, miRNA-coated beads preincubated with AGO2 (Piwi) and/or sNRP1 were even more actively internalized than beads conjugated with naked miRNA alone (Figures 4D and 4E).

These experiments demonstrate that NRP1 and AGO2 bind to each other irrespective of the presence of miRNA. Furthermore, it appears that NRP1/AGO2 complexes retain attached miRNA, and can internalize the bound miRNA. It is important to note that NRP1 can bind to itself, as mentioned previously, and we postulate that extracellular NRP1/AGO2/miRNA complexes have a high affinity for NRP1 on the membrane of cells.

### miRNAs modulate proliferation and migration of NRP1-expressing cancer cells in vitro

**Figure 5:**
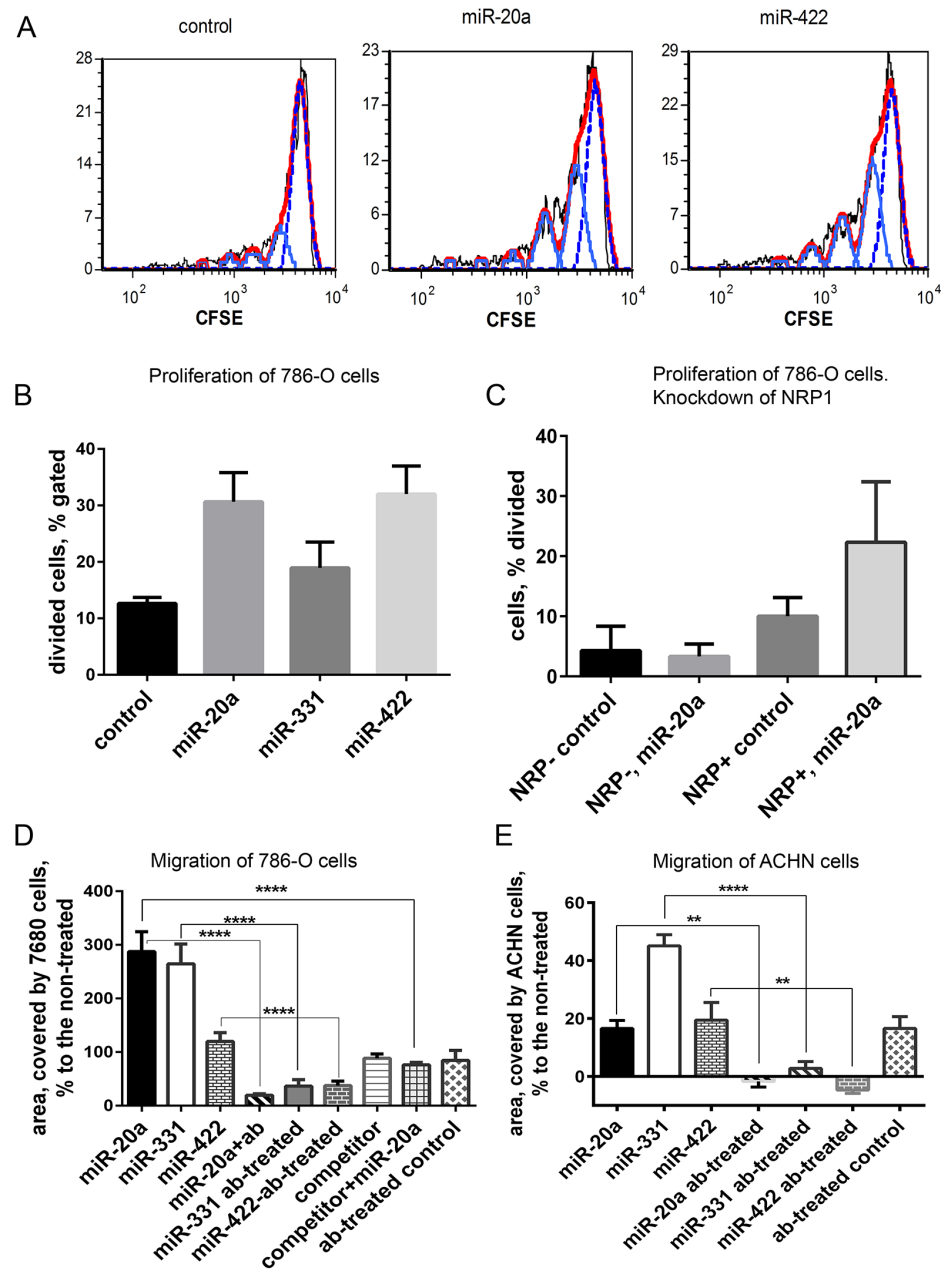
Proliferation and migration of 768-O and ACHN cells is regulated by miRNA mimics **A.** 768-O cells pre-loaded with CFSE and treated with miR-20a or miR-422. Cell division was traced by flow cytometry 24 h later. Generations of divided cells are depicted light blue, undivided cells are shown with dotted blue line, red is calculated fit line. **B.** Division of 768-O cells is activated by miR-20a, miR-331, and miR-422. **C.** Prior knockdown of NRP1 with siRNA abrogated the response of 786-O cells to miR-20a in the proliferation assay. **D.** Migration of 768-O cells pre-treated or not with anti-NRP1 antibody (ab) and treated with miR-20a, miR-331, miR-422, or competitor RNA in the wound-scratch assay. In case of the miRNA competition the cells were pre-treated with the competitor 30 min before adding the equal amount of miR-20a. The data is acquired after 20 h and presented as relative area covered by the migrating cells: A_r_=100×(A_treated_-A_non-treated_)/A_non-treated_. **E.** Migration of ACHN cells treated as in D. The data is representative of three independent experiments. The data (panels B-E) is presented as Mean±SEM, and statistical significance is denoted as: * P<0.05; ** P<0.01; **** P<0.0001.

RNA internalized by a cell via an NRP1-dependent mechanism may induce specific modulation of the cell function. We studied the effect of miRNAs on the proliferation and migration of two renal clear cell carcinoma cell lines, 768-O and ACHN. Importantly, no transfection reagents were added. Three miRNAs (miR-20a, miR-331, and miR-422) increased the division of 768-O cells two-to-three times compared to the negative control (Figure 5A, 5B). Pre-treatment with the blocking anti-NRP1 antibody prevented this miRNA-mediated effect, but for unknown reasons the antibody alone increased proliferation, rendering interpretation difficult (not shown). To demonstrate a role of NRP1, we compared the response of 786-O cells with or without prior siRNA knockdown of NRP1 (Figure 5C). In the experiments presented in Figure 5 and Supplementary Figure S1 (cell proliferation and migration), unlike all other figures, we used a transfection agent (Lipofectamin 3000) to obtain maximal siRNA knockdown of NRP1, which was later removed by multiple washes. 72 h after NRP1 knockdown the cells were incubated with miRNA without the transfection reagent, using unassisted uptake. As can be seen, the NRP1-positive cells had a moderately higher basal rate of proliferation and these cells responded to miR-20a by increased proliferation, whereas those with NRP1 knockdown did not. These results show that the uptake of miRNAs was productive, altering proliferation, and support the conclusion that NRP1 is required for this response.

In a wound-scratch assay, miR-20a increased the migration rate of 768-O cells by 300% and miR-331 by 200%, while miR-422 was less effective (Figure 5D). Migration of ACHN cells was notably activated by miR-331, but miR-20a and miR-422 were much less effective (Figure 5E). A NRP1 blocking antibody, which had only a mild effect on migration by itself, abrogated the ability of the miRNAs to increase migration (Figure 5D, 5E). Knockdown of NRP1 by siRNA reduced cell migration, but these cells were still capable of migration (Supplementary Figure S1A, S1B). In this case, as with proliferation, NRP1 knockdown abolished the effect of miRNA on migration, as seen in images and corresponding data analysis (Supplementary Figure S1A, S1B). In addition, the effect miR-20a on migration was antagonized by a competitor miRNA, which binds to NRP1 but does not participate in miRNA-specific signaling and does not affect migration (see Methods) (Figure 5D). Taken together, these results indicate that miRNAs internalized in an NRP1-dependent process remain functional and regulate cellular processes.

### miRNA-induced tube formation by HUVEC

**Figure 6:**
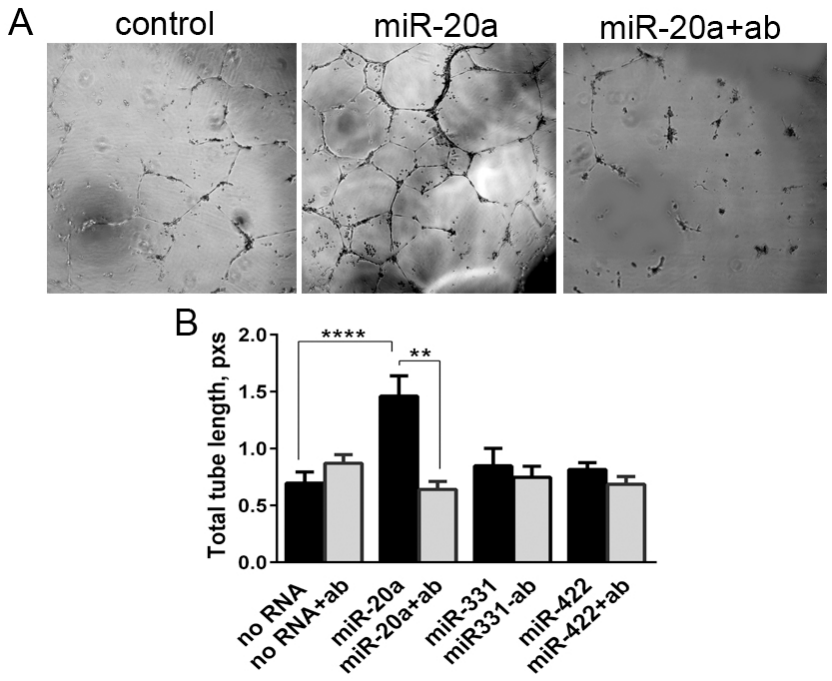
Tube formation assay HUVEC were pre-treated or not with a blocking anti-NRP1 antibody (ab) treated with miRNA mimics, and plated onto Matrigel. The images were taken after 2, 6, and 20 h. The tube formation was quantified as the total length of tubules using ImageJ. **A.** Tube formation by HUVEC treated with miR-20a for 20 h. **B.** Calculated tube length formed by the cells treated with miRNA mimics. The data is presented as Mean±SEM, and statistical significance is denoted as: * P<0.05, ** P<0.01, **** P<0.0001. The data is representative of three independent experiments.

Renal cell carcinoma is a vascular tumor. It was recently postulated that miRNAs released by cancer cells can be an instrument in their cross-talk with both tumor and non-tumor cells, such as vascular endothelial cells [7]. To recognize this signal the recipient cells must express the receptor for miRNA. Human endothelial cells HUVEC cultured on Matrigel can be stimulated to form tubules in vitro. We used this model to show that they can be stimulated with extracellular miRNAs. HUVEC, pretreated or not with the blocking anti-NRP1 antibody, were incubated with miRNAs from our panel. Of the miRNAs tested miR-20a, but not miR-331 and miR-422 mimics activated tube formation (Figure 6A). This selective activation by miR-20a was completely canceled by the NRP1 antibody (Figure 6B). This suggests that unassisted neuropilin-mediated uptake of miR-20a was able to induce specific response in vascular endothelial cells.

## DISCUSSION

In this study, we identify NRP1 as a high affinity receptor for extracellular miRNAs. Moreover, we show that NRP1 internalizes miRNA in either naked form, complexed to beads, or bound to AGO2. The internalized miRNAs exert their usual function.

The presence of miRNA in biological fluids is well established. However, the origin of these miRNA species and their possible function are not clear. They may be released from dead cells with no specific function [2]. They can also be actively secreted [5] and have been postulated to be mediators for intercellular communication (review in [2]). If extracellular miRNA is in fact a signaling agent, we postulate it must be recognized by a receptor on the recipient cell, to induce a specific response. Unassisted productive oligonucleotide uptake by some cell lines (but not others) has been reported [3], but the actual mechanism of cell penetration remains unknown. Indirectly, miRNA pre-bound to HDL can be translocated by the HDL receptor SR-BI [4], but the protein that is binding miRNA was not identified, and the amount of miRNA bound to HDL was only 8% of total circulating miRNA. This route is not active in endothelial cells or peripheral blood mononuclear cells, and its role in the intercellular communication between tumor, endothelium, and immune system appears unlikely [20].

Here, we demonstrated direct binding of synthetic miRNAs to immobilized NRP1 and calculated the Kd values. The affinity was notably increased in the presence of magnesium, and this implies that the RNA affinity to NRP1 is not solely based on the electrostatic interactions, because divalent cations neutralize the negative charge of phosphate groups. This is further confirmed by the observation that a positively-charged polypeptide, poly-ornithine, binds miRNA with a much lower affinity of Kd=6.668±1.293 nM.

We observed no notable difference in retention of miR-331on the plate coated with either full-length NRP1-Fc or the truncated sNRP1 lacking the C-domain, and it appears the a1a2 and b1b2 domains are more essential for the binding than the C-domain. Furthermore, a blocking antibody against the b1b2 domains prevented miRNA binding, suggesting that the principal binding site is located in that region, but further investigation is required to confirm this. The binding curve fits a single-site model; however, this does not exclude multiple non-interacting sites with similar affinity. Retained miRNA was displaced by heparin. This raises the possibility that 20 base-long miRNAs interact with the same sites as heparin. Alternatively, heparin may induce a conformation of NRP1 not favoring the binding of RNA. The identification of binding sites for miRNA requires further studies.

RNA-protein interactions have often been studied in the absence of divalent cations using denatured RNA [21, 22]. Under these conditions oligonucleotide molecules are unfolded and their net negative charge is unmasked. This favors binding of RNA to electropositive pockets of proteins irrespective of RNA conformation. However, these conditions are unmatched in vivo. Here we used physiologically relevant concentrations of calcium and magnesium (0.9 mM Mg^2+^ and 1.26 mM Ca^2+^) and non-denatured miRNA. Thus far, all the miRNAs we have tested bind to NRP1; however, a more exhaustive study is required to identify differences in binding affinities, or miRNA species that do not bind.

The affinity of miRNA to NRP1 may be modulated by the presence of the other ligands of this protein, such as VEGF. We found that VEGF did not notably change the affinity of miRNA to NRP1 and, similarly, miRNA did not alter the affinity of VEGF to NRP1. Recently, Fisher et al. reported that the interaction of VEGF with NRP1 was increased in a narrow range of concentration of total RNA [21]. Their observations cannot be compared directly with our data, because the conditions used in the two studies are markedly different.

While the binding of miRNAs to a recombinant protein in a cell-free assay is an important phenomenon, it is essential to show that the same interaction plays a physiological role in productive translocation of extracellular miRNA. To demonstrate binding and uptake, we conjugated biotinylated miRNA to streptavidin-coated fluorescent microparticles and incubated them with cultured NRP1-expressing cells of the ACHN renal clear cell carcinoma cell line. The treated cells were fixed in paraformaldehyde and cytoplasmic location of the particles was demonstrated by confocal microscopy. It is important to note here that unlike methanol, paraformaldehyde fixation does not induce translocation of the surface material into the cytoplasm as an artifact of fixation process.

Although limited non-specific uptake of the particles was apparent, the number of the particles bound and internalized by the cells was dramatically increased by their conjugation with biotinylated miRNA. In a cell-free assay, the beads without miRNA did not bind to NRP1 more than to a BSA-coated control plate, but binding to NRP1 was increased in the presence of biotin-conjugated miRNA. Particle-labeled miRNA co-localized with NRP1 both on the cell surface and in the cytoplasm. Indeed, more than 85% of the particle-associated green fluorescence was co-localized with NRP1-associated fluorescence signal. NRP1-blocking antibody eliminated the miRNA-mediated increase the green fluorescence in cytoplasm. A small fraction of ACHN cells was naturally NRP1-negative, and they did not uptake the particles. Moreover, siRNA knockdown of NRP1 greatly reduced bead uptake. Therefore, NRP1 binds extracellular miRNA and assists its translocation across the cell membrane. The intracellular fraction of miRNA was notably high compared to the surface-bound fraction.

The remarkable stability of miRNA in the circulation is largely attributed to complex formation with the RNA-binding protein AGO2 [17]. This complex restricts the access of RNases to the RNA backbone. We directly compared the binding of miRNA to AGO2 and NRP1, and found that NRP1 had even higher affinity for miRNA than AGO2. The large size of AGO2 might prevent binding of miRNA to NRP1. To address this question, we performed binding assays and found that AGO2, either in truncated (Piwi domain) or full-length forms, directly binds to NRP1 in the presence or absence of miRNA. Indeed, miRNA did not appear to alter the affinity of the AGO2/NRP1 interaction. Furthermore, our results suggest that AGO2 and NRP1 molecules, when attached together, efficiently retain miRNA. Functionally, the interaction between AGO2 and NRP1 on the cell membrane may facilitate or impede the uptake of miRNA by the cells. To demonstrate that interaction of miRNA with AGO2 does not prevent uptake, we pre-treated biotinylated miRNA with equimolar concentrations of AGO2, sNRP1, or their combination, and conjugated them with the fluorescent streptavidin beads. The uptake by ACHN cells was robust in all three cases. Furthermore, approximately 90% of miRNA pre-treated with AGO2 colocalized with NRP1. Because soluble NRP1 and AGO2 are present in the circulation and body fluids, the formation of AGO2/NRP1/miRNA complexes appears highly probable. Because NRP1 binds to itself, these complexed could have very high affinity for membrane-bound NRP1. In the case of cancer, such complexes may form in the circulation, because these patients have significantly elevated levels of circulating truncated NRP1 [23, 24].

Although translocated into the cytoplasm, miRNA may remain nonfunctional, if it is degraded, trapped in an intracellular organelle, or masked by a protein. To demonstrate productive translocation, we studied the proliferation and migration of 768-O and ACHN cells, treated or not with various miRNA mimics from our panel. In NRP1-expressing cells, but not cells subject to siRNA knockdown of NRP1, unassisted miRNA uptake significantly modulated proliferation. This occurred in a miRNA species-dependent and cell-dependent way. Similar results were obtained in a cell migration assay. For example, treatment with miR-20a increased migration, and this effect was neutralized by a competing miRNA that does not affect migration by itself. Importantly, in the presence of NRP1, but not when it was blocked by antibody or knocked down by siRNA, the miRNAs exerted their expected effects by increasing the rate of migration. Taken together, these findings suggest that the observed effects on proliferation and migration are the result of canonical miRNA signaling. Although most of the cell-bound miRNA is found in cytoplasm, a small fraction of it is membrane-bound, and theoretically it can act in a noncanonical way. This requires further investigation.

Because NRP1 interacts with several growth factors and other ligands, its blockade can alter responses in tumour-cell assays. However, our results with siRNA knockdown strongly support productive internalization of the miRNAs. With this approach, NRP1-low cells are tested with our without miRNA, and can be compared to NRP1-high cells irrespective of other factors. Our results show that NRP1-low cells did not respond to miRNAs. Moreover, in the bioassays we report, the NRP1 blocking antibody effect (without miRNA) was modest and insufficient to alter the interpretation of the results. These findings demonstrate that NRP1 was required to internalize the miRNAs such that they exerted their functions.

Although identification of the miRNA targets is beyond the scope of the current study, our observations match the published data by others. Thus, miR-422 was associated with relapse-free survival of the patients with hepatocellular carcinoma [25] and conceivably may not be a likely activator of migration of cancer cells. miR-20a, a member of the miR-17-92 cluster, activates proliferation of renal clear cells carcinomas [18] and our observations are consistent with the previous report. Since miR-20a targets TGFβ1 signaling pathways and regulates activation of SMAD2/3, it may activate epithelial-to-mesenchymal transition (EMT) and facilitate the migration. The role of miR-331 in various tumors has been studied in details. Overexpression of miR-331-3p induced EMT, activated Akt and growth factor signaling, upregulated expression of EGFR and HER2 in various tumors [26]. It correlated with poor response to therapy and shorter survival time for acute myeloid leukemia patients [27]. Thus, its ability to activate migration of ACHN cells is not surprising. The reported effects of this miRNA species were organ- and tumor type-specific, and this also correlates with our findings.

The mechanisms by which NRP1 induces endocytosis of ligands have not been completely elucidated, but two potential pathways have been described. NRP1 has a cytoplasmic C-terminal motif (SEA) that binds to the PDZ protein synectin (also denoted GIPC) [28, 29]. It has been proposed that once synectin is engaged by NRP1 (and associated ligands) it can act in conjunction with Dab2, to link endosomal vesicles of the clathrin pathway with the actin-based molecular motor myosin VI (Myo6). Myo6 drives endosomal traffic along actin fibers inside the cell [28, 30]. Peptides with a consensus C-end rule (CendR) motif (R/K-X-X-R/K) bind to the same site as VEGF on NRP1 (b1 domain), and are efficiently internalized [11]. Recently, Pang et al. studying CendR NRP1-binding peptides reported that NRP1-mediated endocytosis is different from other endocytic pathways [31]. Interestingly, these authors showed by ultrastructural analysis that CendR peptides and attached cargo were internalized by a process similar to macropinocytosis, although it differed in some mechanistic aspects. Ligands were engulfed in endosome-like vesicles, or more often in structures that had the features of multi-vesicular bodies (MVBs). Interestingly, this pathway was also dependent on an NRP1/synectin interaction, but not on clathrin. These authors suggested this macropinocytosis-like pathway was a bulk mechanism involved in nutrient transport and, indeed, it was stimulated by nutrient depletion. Remarkably, engulfment of NRP1-bound cargo in this pathway led to inter-cellular transfer. Interestingly, Wang et al. reported that NRP1 facilitated the entry of EBV virus into cells through both macropinocytosis and lipid raft-dependent endocytosis [32]. Whether the miRNAs are internalized by a bulk macropinocytosis or other pathway is unknown, and requires further investigation. In the case of AGO2, it binds to NRP1 at high affinity but does not have a CendR motif, similarly to other ligands such as TGF-β1 and its receptors [10], and whether it is internalized by the CendR pathway is also unknown.

In conclusion, NRP1 binds miRNA with high affinity, and is able to translocate its cargo across the cell membrane. Furthermore, AGO2/miRNA complexes are also bound and internalized. The internalized miRNAs preserve their function (productive transfer). Signaling through this mechanism is limited to NRP1-positive cells. It is possible that some selectivity occurs because miRNA species vary in their affinity for the receptor, and this warrants further investigation. Moreover, the response is likely to be cell-type dependent. NRP1 is expressed by many cell types, and we postulate NRP1-dependent internalization of miRNAs has an important physiological function.

## MATERIALS AND METHODS

### MATERIALS

miRNA mimics were purchased from Life Technologies (Thermo Fisher Scientific). Recombinant full-length rat neuropilin-1/Fc chimera (NRP1-Fc), truncated human neuropilin-1 (sNRP1, 1-640), IgG-Fc portion, and VEGF165 were from R&D Systems (Minneapolis). NRP1-Fc has unmodified extracellular domain, while transmembrane and cytosolic domains are replaced with a C-terminal portion of human IgG-Fc. Nuclease-free BSA was from the New England Biolabs (Whitby, Canada). Nuclease-free water was used for solutions (Wisent, Montreal, Canada). NUNC ELISA plates (Thermo Scientific) and Luminata™ Forte ELISA HRP Substrate (Millipore (Etobicoke, Canada)) were used for the luminometric binding assay. ProActive^R^ streptavidin coated fluorescent microparticles labeled with Dragon Green (mean diameter 0.22 μm) were from Bangs Laboratories, Inc. (Fishers, IN, USA). Anti-NRP-1 antibody (MAB 5661, R&D System) home-labeled with AlexaFluor 647 was used for flow cytometry analysis and cell imaging. Additionally, to block NRP1--dependent responses, we used monoclonal antibody MNRP1685A, which is an anti-human/murine NRP1 obtained from Genentech, which binds to the b1b2 domain of NRP1. Phalloidin labeled with AlexaFluor 568 (Molecular Probes (Thermo Fisher Scientific), and DAPI were used for counter-staining in the internalization experiments. CytoFix-CytoPerm kit from BD Biosciences (Mississauga, Canada) was used to fix and permeabilized cells. Cell Trace CFSE cell proliferation kit for flow cytometry was from Life Technologies. Matrigel matrix basal membrane growth factor-reduced was from Corning (Corning, USA). NRP1-targeting and scrambled siRNA were from Santa Cruz Biotechnology. Lipofectamin 3000 (Life Technologies) was used for the knockdown, as indicated. Human recombinant AGO2 (Piwi domain and a full-length protein) was from Biorbyt and anti-pan-AGO2 antibody was from EMD (Etobicoke, Canada)

### Biotinylation of miRNAs at 3′-end

It was performed using Pierce™ RNA 3′ biotinylation kit from Thermo Scientific, according to the manufacturer's protocol. It is based on the ligation of biotinylated cytidine (bis)phosphate to the 3′-end of oligonucleotides. This modification is not expected to affect the binding affinity of miRNA, because it leaves the 5′-end and the seed unmodified. Non-labeled RNA control from the kit

(5′-CCUGGUUUUUAAGGAGUGUCGCCAGAGUGCCGCGAAUGAAAAA-3′) was labeled along with the miRNA species and tested in the binding assay or used as an unlabeled competitor RNA. Denaturation of miRNA was performed as described in the same protocol.

### miRNA binding assay

ELISA plates were coated with NRP1-Fc, sNRP1, AGO2, or with IgG-Fc and blocked with nuclease-free BSA. Non-coated wells treated with BSA were used to estimate the non-specific binding. 0.05% TWEEN in nuclease-free water was used as a wash buffer. Biotinylated miRNA was either denatured at 85°C for 8 min or used undenatured. Binding of miRNA was performed either at 4°C overnight or for 2 h at 37°C. The binding buffer was sterile PBS with or without 0.5 mM MgCl_2_ or sterile nuclease-free HBSS with calcium *(1.26 mM)* and magnesium *(0.9 mM)*. The plate was incubated with streptavidin-peroxidase (R&D Systems) for 20 min. After the wash the plate was kept in the dark for 20 min before the substrate was added in the dark room to minimize auto luminescence. The plate was read using a SpectraMax 5M luminometer-plate reader. The signal integration time was 500 ms. The signal was stable within at least 10 min. Specific binding was calculated by subtraction of the values for the non-specific binding from total binding (all expressed in relative luminescence intensity units, RLU, and denoted as Arbitrary units).

### Microbead binding assay

To examine whether fluorescent streptavidin-coated microbeads used in some experiments had affinity for NRP1-Fc or NRP-Fc/miRNA, plates were coated with NRP1-Fc, or BSA alone, as described above. These plates were incubated, or not, with biotin-conjugated miRNA, and then incubated with the fluorescent streptavidin-coated microbeads with shaking for 20 min. In this case, the beads were resuspended in 0.05 % TWEEN in PBS at 1:1000 ratio and added to the black ELISA plate containing immobilized proteins, with or without retained biotinylated miRNA. The fluorescence was read using ELISA reader with 480 nm excitation and 520 nm emission wavelengths.

### Competition tests

To study the effect of VEGF on the binding of miRNA, the wells coated with sNRP1 and blocked were pre-treated with 1 nM recombinant VEGF for 1 h at room temperature. miRNA was added after wash-out of the unbound VEGF and incubated for 2 h at 37°C. We tested the effect of AGO2 on the miRNA retention by NRP1 and the effect of NRP1 on the miRNA binding to AGO2 in a similar way. Equimolar mix of AGO2 and miRNA in Ca/Mg-HBSS was incubated for 1 h at 37°C and diluted serially for the binding assay. The detection of the bound miRNA was performed as above.

### Protein binding assays

To study the effect of miRNA on the binding of VEGF a plate was coated with sNRP, blocked, and pre-treated with miRNA for 2 h before adding VEGF. The bound VEGF was detected with anti-VEGF primary antibody (R&D Systems) and secondary anti-mouse IgG-HRP (Promega) with TMB substrate. Binding of AGO2 to NRP1-Fc was studied in a similar way. In addition, equimolar mix of AGO2 and miRNA in Ca/Mg-HBSS was incubated for 1 h at 37°C and diluted serially for the binding assay to study the binding of the AGO2-miRNA protein complex to NRP1. Protein retention was quantified using anti-pan AGO2 primary antibody (EMD) and secondary anti-mouse IgG-HRP (Promega (Madison, USA) with TMB substrate. The binding was expressed in arbitrary units defined as OD450, after the subtraction of the non-specific binding.

### Cell culture

Renal Clear Cell Carcinoma cells 768-O and ACHN were grown in RPMI-1640 supplemented with 10 % FBS. HUVEC cells were grown in F12K supplemented with ECGs (0.75 mg/ml; Sigma), heparin (0.1 mg/ml) and 10 % FBS. BT-474 cells were grown in DMEM, supplemented with 10% FBS. For loading with miRNA cells were harvested with Ca/Mg-free HBSS+5 mM EDTA. 1.5×10^4^ cells were resuspended in serum-free medium containing 1 mg/ml RNAse-free BSA and incubated with 5 pmol miRNA in a total volume of 300 μL for 30 min at 37°C with periodic gentle mixing. After the incubation they were plated to be used in the proliferation or wound-scratch assays.

### RNA internalization assay

ACHN cells were seeded onto the chamber-slide at 2×10^4^ cells per well. Before the assay the cells were rinsed with the serum-free medium and pre-treated or not with blocking anti-NRP1 antibodies (20 μg/ml each) for 30 min in the incubator. In some cases miRNA was pre-treated with 50 nM of either sNRP1 or recombinant AGO2 (as indicated in the legends). For the conjugation, 5 pmol of biotinylated miRNA were mixed with 1-10 μl of the fluorescent streptavididin-coated microparticles and 1 mg/ml BSA in total volume of 20 μL. The mixture was vortexed for 15 min at room temperature and low speed, protected from light, as recommended by the manufacturer of the beads. After the conjugation step miRNA was diluted with serum-free BSA-containing medium (1 mg/ml) to the final concentration of 16 pM and loaded onto the pre-treated cells for 30 min at 37°C. After the incubation the cells were rinsed in serum-free medium, fixed in paraformaldehyde, permeabilized, and counter-stained with anti-NRP1-AlexaFluor 647, phalloidin-AlexaFluor 568, and DAPI. The slide was mounted in ProLong Gold (Molecular Probes) and analyzed using Zeiss LSM700 confocal microscope with 63x magnification and z-sections with 1μm interval. Co-localization of the microparticles and NRP1 was estimated using ImageJ. RNA uptake was estimated as an average fluorescence intensity of the fluorescent beads per cell area delineated by F-actin staining, in z-stacks.

### Knockdown of NRP1

Unassisted translocation of siRNAs was demonstrated in the NRP1-expressing BT-474 cells as following: NRP1-targeting siRNA or scrambled siRNA (Santa Cruz Biotechnology) was added to the cultured cells in serum-free medium for 4 h before replacing it with DMEM, containing 10 % FBS. 72 h later the cells were harvested, fixed, permeabilized, and stained against NRP1. Total expression of this protein was evaluated by flow cytometry. Similarly, knockdown of NRP1 in 786-O and ACHN cells was achieved by the unassisted uptake of the siRNA, and the 10^5^ cells were incubated with 60 pmol of siRNA in serum-free medium for 30 min at 37°C.

In the experiments of renal cell carcinoma proliferation and migration only, for maximal knockdown of NRP1, we used Lipofectamin 3000 and the same amount of siRNA as for the unassisted transfection, and a protocol described by the manufacturer.

### Proliferation assay

Renal Clear Cell carcinoma 768-O cells were harvested with HBSS-EDTA, rinsed in serum-free RPMI 1640. 10^6^ cells were mixed with 1.2 μl CFSE in 1 ml of the same medium and incubated for 20 min at 37°C. After rinsing in RPMI-10 % FBS and serum-free RPMI, 3×10^4^ CFSE-labeled cells and 10 pmol miRNA mimic were resuspended in 600 μl of the serum-free medium for 30 min at 37°C. After the incubation and loading with miRNA (16 pM, 30 min, 37°C), with or without the pre-treatment with the NRP1-blocking antibodies, they were transferred into the complete growth medium and plated. They were analyzed by flow cytometry after 24 h or 48 h in culture and staining with propidium iodide. Proliferation was quantified as a % divided cells in FCS Express 4.

### Migration assay

ACHN and 768-O cells were harvested with HBSS-EDTA, pre-treated or not with the anti-NRP1 antibody or a competitor miRNA (as indicated), loaded with miRNA mimics in serum-free medium as above and grown to 80% confluence in 24 well plates. The competitor RNA was used in the same concentration as the tested miRNA and was pre-loaded 30 min before miRNA was added. The wound was scratched with a 200-μl tip, and the area covered by migrating cells was calculated at various time points using ImageJ.

### Tube formation assay

96-well plate was coated with 50 μl Matrigel diluted with unsupplemented F12K medium 1:1. HUVEC (passages from 6 to 12) were pre-treated or not with NRP1-blocking antibodies and loaded with miRNA as described above. They were plated onto the Matrigel-coated plate at 15000 cell per well. The cells were photographed after 2, 6, and 20 h. The total length of the formed tubes was calculated in ImageJ.

### Statistical analysis

Statistical analysis was performed in Prism 6 GraphPad Software using ANOVA. P value ≤0.05 was considered statistically significant.

## SUPPLEMENTARY MATERIALS FIGURE


