# Supplementary material for: Neuropilin-1 is a receptor for extracellular miRNA and AGO2/miRNA complexes and mediates the internalization of miRNAs that modulate cell function
Source: Oncotarget. 2016 Jul 29;7(42):68057–71. doi: 10.18632/oncotarget.10929 (PMC5356539; doi:10.18632/oncotarget.10929)
Supplement: Supplementary file 1 [file oncotarget-07-68057-s001.pdf]

Neuropilin-1 is a receptor for extracellular miRNA and AGO2/miRNA complexes and mediates the internalization of miRNAs that modulate cell function

SUPPLEMENTARY FIGURE

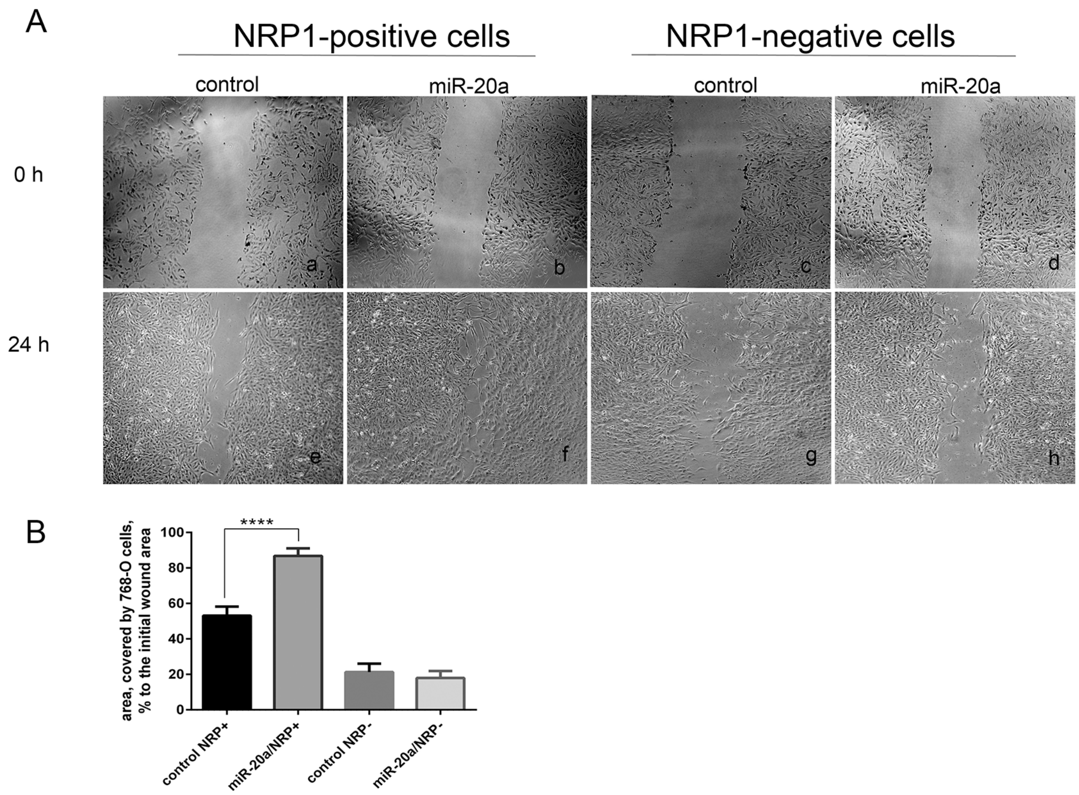

Supplementary Figure S1: NRP1 mediates the unassisted uptake of miR-20a by 786-O cells and the activation of their migration by this miRNA. A. To knockdown NRP1 786-O cells were transfected with either scrambled siRNA (a, b, e, f) or NRP-targeting siRNA (c, d, g, h), using Lipofectamin 3000. 72 h later they were treated (b, f, d, h) or not (a, c, e, g) with miR-20a using the unassisted uptake, and wounds were scratched with a 200 uL pipette tip. The images of the wounds were acquired immediately (time 0 h) and 24 h later using Nikon eclipseTS100. The images were analyzed in ImageJ. **B.** Area covered by the cells is presented as % of the initial wound area. Activation of the migration of 786-O cells by miR-20a was significant ($p<0.0001$) only in the NRP1-positive cells, but not in the NRP1-negative cells.
